# Enhancement of Solubility, Stability, Cellular Uptake, and Bioactivity of Curcumin by Polyvinyl Alcohol

**DOI:** 10.3390/ijms25116278

**Published:** 2024-06-06

**Authors:** Smee Kang, Minkyoung Kim, Hyelin Kim, Jungil Hong

**Affiliations:** Department of Food Science and Technology, College of Science and Convergence Technology, Seoul Women’s University, 621 Hwarang-ro, Nowon-gu, Seoul 01797, Republic of Korea; michael1224@hanmail.net (S.K.); rlaalsrud080@swu.ac.kr (M.K.); hyei316@swu.ac.kr (H.K.)

**Keywords:** curcumin, polyvinyl alcohol, photostability, solubility, cytotoxicity, cellular uptake, aqueous system

## Abstract

The biological activities and related mechanisms of curcumin, a major polyphenolic compound in turmeric, the rhizome of *Curcuma longa*, have been extensively investigated. Due to its poor solubility in water, the analysis of curcumin’s biological activities is limited in most aqueous experimental systems. In the present study, the effects of polyvinyl alcohol (PVA), a dietary-compatible vehicle, on the solubility, stability, cellular uptake, and bioactivities of curcumin were investigated. Curcumin solubility was improved significantly by PVA; the color intensity of curcumin aqueous solution in the presence of PVA increased concentration-dependently with its peak shift to a shorter wavelength. Improved suspension stability and photostability of curcumin in an aqueous solution were also observed in the presence of PVA, even at 62.5 μg/mL. The scavenging activities of curcumin against DPPH, ABTS, AAPH radicals, and nitric oxide were enhanced significantly in the presence of PVA. PVA at 250 μg/mL also significantly enhanced the cytotoxic activity of curcumin against both HCT 116 colon cancer and INT 407 (HeLa-derived) embryonic intestinal cells by reducing the IC_50_ from 16 to 11 μM and 25 to 15 μM, respectively. PVA improved the cellular uptake of curcumin in a concentration-dependent manner in INT 407 cells; it increased the cellular levels more effectively at lower curcumin treatment concentrations. The present results indicate that PVA improves the solubility and stability of curcumin, and changes in these chemical behaviors of curcumin in aqueous systems by PVA could enhance the bioavailability and pharmacological efficacy of curcumin.

## 1. Introduction

Curcumin is a yellow polyphenolic pigment obtained from turmeric (*Curcuma longa* Linn), a member of the ginger family which is native to eastern India and is one of the major curcuminoid compounds, along with demethoxycurcumin (DMC) and bisdemethoxycurcumin (BMC) [1,2]. Traditionally used as a coloring agent and spice in various cuisines including curries, curcumin has extensive applications in dietary supplements, cosmetics, and pharmaceuticals [3]. Many studies have documented the diverse biological activities of curcumin, including its antioxidant [4,5], anti-inflammatory [5,6,7,8], and anti-cancer [8,9,10,11,12] properties and its preventive activity against Alzheimer’s disease [13,14]. In addition, curcuminoids exhibit various health benefits for the skin, such as thwarting skin-cell oxidation, averting UV irradiation-induced cytotoxicity, and inhibiting the microphthalmia-associated transcription factor (MITF) and tyrosinase activities involved in skin pigmentation processes [15,16]. Curcuminoids, including curcumin, DMC, and BMC, have also been reported to confer many other beneficial health effects [17].

Curcuminoids consist of two ferulic acids linked in an α, β-unsaturated diketone structure that is poorly soluble and chemically unstable in aqueous solution. Curcumin exists in a keto form under acidic and neutral pH storage conditions. Under alkaline conditions above pH 8, it converts to the enolate form, and its stability is greatly reduced [18,19,20]. Lee et al. [19] reported that the yellow color intensity of curcumin decreases as the pH increases. Curcumin is also easily hydrolyzed to ferulic acid, vanillin, feruloyl methane, and other smaller molecules in aqueous solution [21,22]. These chemical properties make it difficult to accurately measure the bioactivities of curcumins in various in vitro hydrophilic experimental conditions, including cell culture systems. Due to its hydrophobic nature, curcumin is poorly dispersed in the intestinal tract, which greatly reduces its absorption through enterocytes [2]. Therefore, there is a growing need for research on delivery systems to increase curcumin bioavailability and on delivery vehicles to more accurately assess the curcumin activity in aqueous experimental systems.

Various polymeric compounds, nanomaterials, and hybrid delivery systems have been investigated as a way to increase the aqueous dispersibility and bioavailability of poorly soluble drugs and hydrophobic dietary ingredients such as curcumin [14,15,23,24,25,26]. Several polymers, including proteins, polysaccharides, polyvinyl alcohol (PVA), poly (lactic-co-glycolide) (PLGA), and polyethylene glycol (PEG), have been widely used as a carrier vehicle in the pharmaceutical and food industries [26,27,28]. PVA is a non-toxic, water-soluble, and dietary applicable polymer that has been used as a stabilizer and a biofilm material due to its high mechanical strength and good biocompatibility [27,29]. However, little research has been conducted on the application of PVA to pharmaceutical and food products. Furthermore, the application of polymeric compounds such as PVA as a vehicle for measuring the bioactivity of curcumin, which is poorly soluble in water and therefore incompatible with hydrophilic experimental systems, has not been reported before.

This study investigated the effects of PVA on the chemical stability, dispersion stability in solution status, and bioactivities, including antioxidant activity and cytotoxicity, as well as the cellular uptake of curcumin. This study aimed to demonstrate that PVA can contribute to more accurate activity analysis by providing a suitable environment for the activity expression of hydrophobic compounds such as curcumin in hydrophilic experimental environments, including cell culture systems. In addition, we also sought to provide information on the potential use of PVA as a biocompatible material to enhance the absorption and bioavailability of curcuminoids for the development of curcumin-related products.

## 2. Results and Discussion

### 2.1. Fluorescence and Absorbance Properties of Curcumin in PVA Solution

**Figure 1:**
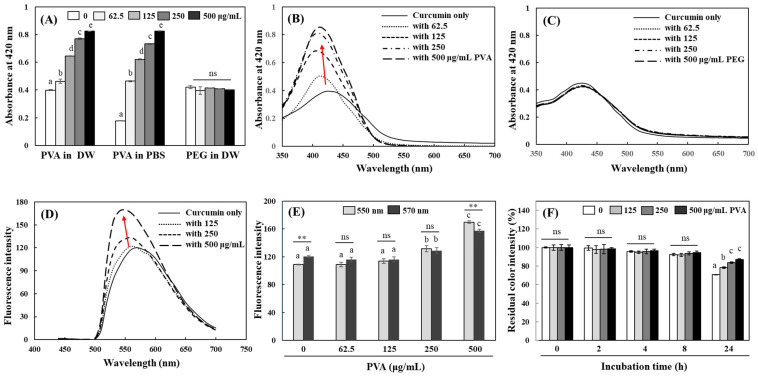
Effects of PVA and PEG on absorbance and fluorescence properties and stability of curcumin. Peak absorbance changes of curcumin (40 μM) in DW and PBS in the presence of different concentrations of PVA and PEG (0–500 μg/mL) were measured (**A**). The absorbance spectra of curcumin (40 µM) dissolved in DW in the presence of different concentrations of PVA (**B**) and PEG (**C**) (0–500 μg/mL) were scanned at 350–700 nm. Changes in fluorescence emission spectrums at 450–700 nm (**D**) and emission fluorescence intensity at 550 and 570 nm (**E**) (Ex. 440 nm) of curcumin dissolved in DW by different concentrations of PVA were also analyzed. Curcumin in DW with different concentrations of PVA was incubated at RT in the dark, and the color intensity of curcumin during 24 h was analyzed at 420 nm (**F**). Each value represents the mean ± SD (*n* = 3–6). Different letters indicate a significant difference (*p* < 0.05) and **, significantly different from each other according to Student’s *t*-test (**, *p* < 0.01) in (**E**). ns—not significant.

Curcumin solutions in distilled water (DW) or phosphate-buffered saline (PBS) with different concentrations of PVA were prepared and examined with regard to their absorption and fluorescence properties. The curcumin solution dissolved in DW exhibited a peak absorption wavelength region of around 420 nm, and the peak absorbance of the curcumin solution was significantly increased in the presence of PVA. The color intensity of curcumin was much less pronounced in PBS, probably because PBS contains salt ions that make the solvent more polar. However, the addition of PVA effectively increased the peak absorbance of curcumin in PBS to a level similar to that of curcumin in DW. Another commonly used polymer, polyethylene glycol (PEG), did not affect the curcumin color intensity (Figure 1A). The absorbance spectrum of curcumin solution, which ranged between 350 and 450 nm, was also significantly enhanced according to the increase in the PVA concentration, and a slight blue shift of peak absorbance was observed (Figure 1B). The overall absorbance spectrum of curcumin solution did not show a significant change by PEG (0–500 µg/mL) either (Figure 1C). It was observed that partially hydrolyzed PVA (80% or 87–89%) with lower average molecular weight was more effective at enhancing the color intensity of curcumin is aqueous solution than fully hydrolyzed PVA (>99%). The precise modulatory effects of the degree of hydrolysis and molecular weight of PVA on the solubilization of curcumin need to be further investigated.

The curcumin solution excited at 440 nm exhibited peak emission fluorescence characteristics around 570 nm; a fluorescence spectral change of curcumin solution in the presence of PVA was also observed. The emission fluorescence intensity of curcumin was significantly enhanced in the presence of more than 250 µg/mL of PVA, showing a peak shift to a shorter wavelength (Figure 1D). The original curcumin solution exhibited an emission peak at 570 nm, but as the concentration of PVA in the solution increased, the emission peak shifted to 550 nm; significantly higher fluorescence intensity at 550 nm than 570 nm was observed in a curcumin solution containing 500 µg/mL (Figure 1E). When curcumin solutions containing PVA were stored in the dark, the decrease in curcumin color intensity was significantly improved in a PVA concentration-dependent manner after 24 h storage (Figure 1F).

Curcumin is connected through the β-diketone structure, which induces keto–enol tautomerization conformational changes, and these changes greatly affect the stability of curcumin [30,31]. Solvent polarity is one of the most important factors in the formation of different keto and enol tautomers. The keto–enol equilibrium of curcumin is shifted toward the formation of more keto forms in less hydrophilic solvents that provide greater stability [31]. The blue shift in peak absorbance indicates a transition to a more keto form of curcumin. It was also observed that the broad peak shape of the curcumin spectrum in the 400–500 nm range in DW narrowed and sharpened in the presence of PVA (Figure 1B). The broad spectrum is attributed to the hydrogen bonding of different curcumin tautomers with solvent molecules, implying more enol form formation and less stabilization of curcumin in solution [31,32,33]. The increase in solvent polarity decreased the fluorescence intensity of curcumin and shifted the emission fluorescence peak to a shorter wavelength [28,30]. The blue shift of the emission peak also correlates with the formation of a micellar structure in which curcumin tends to become buried [34]; the changes in the fluorescence property of curcumin by PVA imply that curcumin may be covered by PVA molecules, reducing its exposure to water molecules. The present results suggest that the presence of PVA could reduce the solvent polarity of DW and increase the solubility of curcumin. Accordingly, PVA also improves the stability of curcumin in aqueous systems.

### 2.2. Changes in Photostability by PVA

**Figure 2:**
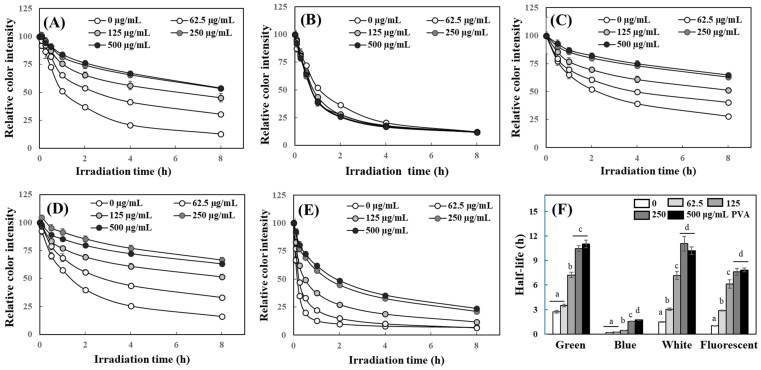
Effects of PVA on the photostability of curcumin under irradiation of different light sources. Curcumin (40 μM) dissolved in DW containing different concentrations of PVA (**A**,**C**–**F**) or PEG (**B**) was irradiated under fluorescent light (**A**,**B**), green (**C**), white (**D**), and blue (**E**) LED (each 10 W/m^2^) for 8 h at RT, and photodegradation of curcumin was detected at 420 nm. The half-lives for the color degradation in the presence of PVA (**F**) were also calculated. Each value represents the mean ± SD (*n* = 4) and different letters indicate a significant difference (*p* < 0.05) in (**F**).

Curcumin is used in a variety of applications, not only as a spice but also as a colorant, so its chemical stability under light is an important issue. In this experiment, the effects of PVA on the photostability of curcumin under different lights were investigated. Exposure to ordinary fluorescent light (10 W/m^2^) induced ~50% photodegradation of curcumin within 1 h in DW; the presence of PAV in the aqueous solution significantly enhanced the photostability of curcumin (Figure 2A). However, PEG did not improve the photodegradation of curcumin under fluorescent light, but rather slightly decreased its stability (Figure 2B). Color degradation of curcumin was also induced by exposure to different LEDs including green, white, and blue LED (10 W/m^2^ each); blue LED showed the strongest effects on curcumin degradation (Figure 2C–E). This may be because the peak emission wavelength of the blue LED (447 nm) is close to the peak absorbance of the curcumin solution. PVA also effectively improved the photostability of curcumin in a concentration-dependent manner. The half-lives for color degradation measured at 420 nm of curcumin dissolved in DW were significantly extended in the presence of PVA under all irradiation lights used in this study (Figure 2F). In general, an increase in color intensity and absorbance value indicates an increase in the amount of light absorbed by a substance. The degradation of a pigment compound can be accelerated by increasing the absorbed light energy. In the present results, the degradation of curcumin was most pronounced under the irradiation of blue LED with a peak emission wavelength (447 nm) closest to the maximum absorption wavelength of curcumin (Figure 2F and Figure S1). However, an improvement in the photostability of the curcumin was observed despite the increase in the absorbance of the curcumin solution in the presence of PVA. This stabilizing effect of PVA was observed not only in the dark, but at most of the visible-light wavelengths used. This is believed to be a result of chemical stabilization which is achieved by reducing the interaction of curcumin with water molecules in an aqueous solution, rather than a simple light-blocking effect of PVA.

Thus, PVA not only enhanced the chemical stability of curcumin in the typical aqueous solution state but also improved its chemical stability more effectively under light-induced photodegradation conditions. This is also thought to be because PVA improves the solubility of curcumin in an aqueous solution and increases its chemical stability by reducing interactions with water molecules.

### 2.3. Changes in Antioxidant Activity of Curcumin by PVA

**Figure 3:**
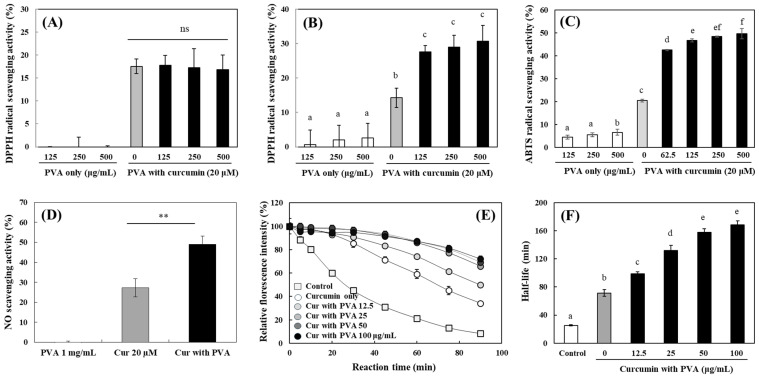
Modulation of antioxidant activity of curcumin by PVA. Effects of PVA on the scavenging activity of DPPH radical of curcumin (20 µM) dissolved in MeOH (**A**) or DW (**B**) were measured. Changes in scavenging effects against ABTS radical (**C**) and NO (**D**) of curcumin in the absence or presence of PVA were also analyzed. The fluorescence intensity of the reaction mixture containing fluorescein, AAPH radical, and curcumin (final 1.25 μM) in the presence of PVA (0–100 μg/mL) was measured at Ex. 485 nm and Em. 535 nm (**E**), and the half-lives for the fluorescence decrease were calculated (**F**). Each value represents the mean ± SD (*n* = 6–9). **—significantly different from the control without PVA according to Student’s *t*-test (**, *p* < 0.01) (**D**). Different letters indicate a significant difference (*p* < 0.05) based on one-way ANOVA and Tukey’s HSD test. ns—not significant.

Curcumin shows an antioxidant property and hydroxyl groups on the phenolic ring are mainly responsible for the activity [4]. Modulation of antioxidant activities of curcumin against various radicals by PVA was investigated in different in vitro assay systems. The 2-diphenyl-2-picrylhydrazyl (DPPH) radical showing a blue-green color is stable in an organic solvent such as methanol (MeOH), and its color has been bleached by the transfer of hydrogen or electrons from reducing agents [35]. The scavenging activities of curcumin dissolved in two different solvents including MeOH and DW against DPPH radical were analyzed after being mixed with an equal volume of DPPH MeOH solution. The radical scavenging activity of curcumin (20 μM) in MeOH and DW was 18 and 15%, respectively, which was slightly stronger in MeOH; PVA did not show considerable DPPH radical scavenging activity in both solvents (Figure 3A,B). While PVA had no effect on the curcumin activity in MeOH, significantly enhanced activity of curcumin in DW was observed in the presence of 125 μg/mL PVA as compared to one without PVA (Figure 3A,B). However, a further increase in the PVA concentration did not result in any further increase in the activity.

Along with DPPH, 2,2′-azinobis-(3-ethylbenzothiazoline-6-sulfonic acid (ABTS) is another radical that is widely used to evaluate the antioxidant activity of many natural products. The ABTS radical exhibits a strong absorption at 734 nm and loses its color by electron donation from antioxidants. Curcumin scavenged ~20% of the ABTS radical at 20 µM, and PVA also showed a further 4–6% inhibitory activity at concentrations of 125–500 µg/mL, with no significant difference depending on the PVA concentration (Figure 3C). The ABTS radical scavenging activity of curcumin was significantly enhanced in the presence of PVA; the PVA-induced increase in the curcumin activity was much greater than the DPPH radical scavenging activity and was PVA concentration-dependent (Figure 3C). The scavenging activity of curcumin against nitric oxide (NO) was also found to be significantly increased in the presence of PVA (Figure 3D). PVA showed no NO scavenging activity even at 1 mg/mL, and curcumin alone exhibited ~27% scavenging activity. The NO scavenging activity of curcumin increased ~2-fold in the presence of 1 mg/mL PVA (Figure 3D). In addition, the activity of curcumin and changes in the activity by PVA were analyzed via the oxygen radical absorbance capacity (ORAC) assay, which is widely used to measure antioxidant activity [36]. In the ORAC reaction system, fluorescence decay of fluorescein by 2,2′-azo-bis(2-aminopropane) dihydrochloride (AAPH) radicals occurs, and the scavenging activity against AAPH radicals is evaluated by measuring how much this decay is inhibited by antioxidants present in the assay system [36,37]. In this ORAC assay system, the activity of curcumin was highly sensitive, significantly delaying the fluorescence decay of fluorescein even at 1.25 μM. PVA markedly enhanced the AAPH radical scavenging activity of curcumin even at concentrations as low as 12.5 μg/mL, and no further increase in the curcumin activity was observed at concentrations above 50 μg/mL (Figure 3E). To compare the changes in curcumin activity at different PVA concentrations, the half-life for the decay of fluorescein fluorescence was calculated in each condition. The results showed that the fluorescein half-life of 71 min for curcumin alone was significantly prolonged to 99 min in the presence of 12.5 μg/mL PVA. The activity of curcumin was enhanced in a PVA concentration-dependent manner up to 50 μg/mL, which exhibited a half-life of 158 min (Figure 3F).

The above results indicate that PVA significantly enhanced the activity of co-existing curcumin despite having no or negligible effect on various antioxidant assay systems. PVA enhanced the activity of curcumin in several antioxidant assay systems; the effects of PVA were more pronounced in aqueous assay systems than in systems using organic solvents such as the DPPH radical scavenging activity assay. Therefore, in an aqueous reaction system where the behavior of curcumin with a hydrophobic nature is limited, PVA appears to play a role in increasing the solubility of curcumin and facilitating its dispersion. This may provide a compatible environment for the expression of curcumin activity.

### 2.4. Effects of PVA on Solution Stability and Physicochemical Property of Curcumin

**Figure 4:**
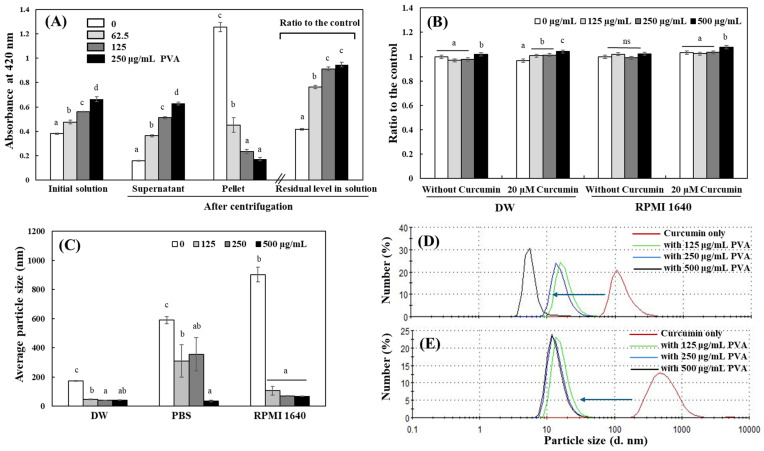
Changes in dispersion stability and physicochemical property of curcumin solution by PVA. The solution stability of curcumin dissolved in DW containing different concentrations of PVA under centrifugal force (14,000 RCF) was analyzed as described in the Materials and Methods section (**A**). Changes in the viscosity of curcumin solution in DW and RPMI 1640 medium by PVA were measured (**B**). The average particle size of curcumin solution in DW, PBS, and RPMI 1640 medium (**C**), and the size distribution of curcumin particle in DW (**D**) and RPMI 1640 (**E**) were also analyzed using a Zeta potential analyzer. Each value represents the mean ± SD (*n* = 3–6), and different letters indicate a significant difference (*p* < 0.05).

It is expected that the enhancement of curcumin activity in the presence of PVA is attributed to the increased solubility of curcumin by PVA in an aqueous system. Subsequent experiments investigated how PVA modulates the physicochemical properties of curcumin solution in different aqueous systems. Since curcumin is poorly soluble in water, it exists as an unstable colloid that clumps together in aqueous solution. Therefore, the suspension stability of curcumin in DW was evaluated based on the level of curcumin separated by applying centrifugal force to the aqueous curcumin solution. After centrifuging the curcumin solution at 14,000 RCF for 10 min, the absorbance of the solution was compared to that before centrifugation; the peak absorbance of the curcumin solution at 420 nm decreased by 58% after the centrifugation. The presence of PVA, however, increased the dispersion stability of curcumin in the aqueous solution. The residual curcumin content based on color intensity after centrifugation of the solution was increased according to the increase in PVA concentrations in the solution. Residual curcumin in the solution was >90% in the presence of 125–250 μg/mL PVA (Figure 4A). On the other hand, the precipitated pellet, i.e., the amount of curcumin that was not stable in the solvent phase and was separated by centrifugation, was measured indirectly by dissolving it with DMSO. The amount of precipitate was inversely proportional to the concentration of PVA (Figure 4A). The results indicate that PVA has a significant effect on increasing the chemical stability of curcumin, curcumin solubility, as well as its suspension stability. Therefore, it is believed that PVA may have a positive impact on the production of more accurate and objective results on the expression of curcumin activity in aqueous experimental systems.

Effects of PVA on the physicochemical properties of different solutions containing curcumin were evaluated. Curcumin solutions in DW, RPMI 1640 cell culture medium, or PBS with different concentrations of PVA were prepared, and their viscosity and colloidal properties were analyzed. The viscosity of DW containing 0–250 μg/mL PVA alone was not changed significantly but slightly increased in the presence of 500 μg/mL. The addition of PVA resulted in a PVA concentration-dependent increase in the viscosity of curcumin-containing DW solution, but the changes did not exceed 4% (Figure 4B). PVA alone did not affect the viscosity of RPMI 1640 culture medium up to a concentration of 500 μg/mL. PVA did not affect the viscosity up to 250 μg/mL in the medium containing curcumin; an increase in viscosity of the medium was observed in the presence of 500 μg/mL PVA (Figure 4B). The addition of polymers such as PVA has been reported to increase the viscosity of solutions [38], and some increase in the viscosity of curcumin solutions was observed due to the addition of PVA in this experiment. However, the increase in viscosity by PVA was within a 4% maximum and the effect of PVA was less pronounced in cell culture media; it is unlikely to have a significant effect on growing cells when PVA is used as a vehicle in cell culture experiments.

Curcumin is thought to exist in a clumped state because it is not efficiently dispersed in aqueous solutions. Therefore, the changes in the dispersion status and particle sizes of curcumin in a solution by PVA were analyzed using a Zeta potential analyzer. The average particle size of the curcumin solution increased in the order of 173, 590, and 903 nm in DW, PBS, and RPMI 1640 medium, respectively (Figure 4C). Thus, curcumin was not in a stable solution in the aqueous solvent; it was mostly aggregated into particles above colloidal size (>100 nm). The addition of PVA significantly decreased the particle size in all solutions. The reduction of particle size in curcumin solution by PVA was particularly pronounced in DW and RPMI 1640 media; a 3.7- and 8.5-fold reduction in average particle size, respectively, was observed even with the addition of 125 μg/mL PVA (Figure 4C). The size distribution of curcumin particle in DW and RPMI 1640 medium also showed a significant decrease in the diameter of the main particles with the increase in PVA concentration. The major particle size of curcumin was reduced to a normal colloidal size state below 100 nm in the presence of 125 μg/mL PVA in either DW or RPMI medium (Figure 4D,E). This result suggests that PVA reduces the size of aggregated curcumin particles by promoting the dispersion of curcumin, which is poorly soluble in water and thus its particles aggregate with each other.

### 2.5. Alteration of the Cytotoxic Effect of Curcumin by PVA

It is interesting to note that the aggregated curcumin particles observed were significantly larger in size in PBS and RPMI 1640 medium compared to DW (Figure 4C). This is probably due to the presence of large amounts of hydrophilic solutes such as salts in the solutions, resulting in the salting-out effect on curcumin. Therefore, in cell culture media with higher concentrations of hydrophilic solutes such as sugars, amino acids, water-soluble vitamins, and salts, the activity of curcumin is likely to be much more limited than in a simple aqueous solution. The following experiments analyzed the effect of PVA on curcumin bioactivity in cell culture systems.

**Figure 5:**
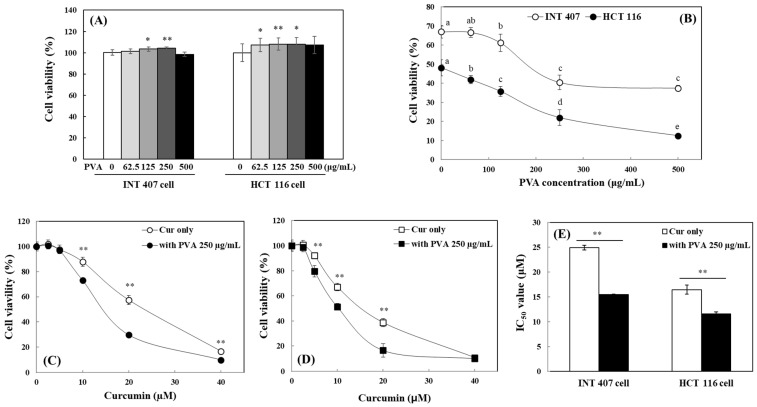
Changes in curcumin cytotoxicity against INT 407 and HCT 116 cells by PVA. Effects of different concentrations of PVA only (**A**) or with 20 μM curcumin (**B**) on the viability of INT 407 and HCT 116 cells were analyzed after 24 h treatment. Concentration-dependent effects of curcumin with or without PVA on viabilities of INT 407 (**C**) and HCT 116 (**D**) cells were analyzed after 24 h incubation, and IC_50_ values were also calculated (**E**). Each value represents the mean ± SD (*n* = 8). *, **, significantly different with and without PVA according to Student’s *t*-test (*, *p* < 0.05; **, *p* < 0.01) (**A**,**C**–**E**). Different letters indicate a significant difference (*p* < 0.05) based on one-way ANOVA and Tukey’s HSD test (**B**).

The modulation of cytotoxic property of curcumin by PVA was evaluated in both INT 407, (HeLa-derived) embryonic intestinal cells, and HCT 116 colon cancer cells. Treatment of INT 407 and HCT 116 cells with PVA alone up to a concentration of 500 µg/mL did not show any toxicity, but rather promoted cell viability up to 7–8% in the range of 62.5–250 µg/mL (Figure 5A). Curcumin exhibited cytotoxicity at a concentration of 20 µM, reducing the viability of INT 407 and HCT 116 cells by 33.0 and 51.9%, respectively. The cytotoxic potency of curcumin against both cell lines was significantly enhanced in the presence of PVA; PVA increased the cytotoxic activity of curcumin in a concentration-dependent manner, and the efficacy was more pronounced in HCT 116 cells (Figure 5B).

Curcumin also induced concentration-dependent cytotoxicity against both INT 407 and HCT 116 cells with higher potency on HCT 116 colon cancer cells (Figure 5C,D). Co-treatment with 250 µg/mL PVA also significantly potentiated the cytotoxic activity of curcumin against INT 407 and HCT 116 cells above 5 and 2.5 µM, respectively. To assess the extent to which the cytotoxicity of curcumin was enhanced by PVA, IC_50_ values (concentrations that the concentration that inhibited cell viability by 50%) were calculated (Figure 5E). In INT 407 cells, the IC_50_ of curcumin was 25 µM, while it was reduced to 15 µM in the presence of 250 µg/mL PVA. IC_50_ was 16 µM in HCT 116 cells when treated with curcumin alone and was significantly reduced to 11 µM in the presence of 250 µM of PVA (Figure 5E). The results indicate that the co-treatment of PVA induces significantly enhanced cytotoxicity of curcumin in both cell lines. This is probably because curcumin is more readily accessible to cells in the presence of PVA, and PVA makes cells respond more sensitively to curcumin.

### 2.6. Changes in Intracellular Uptake of Curcumin by PVA

**Figure 6:**
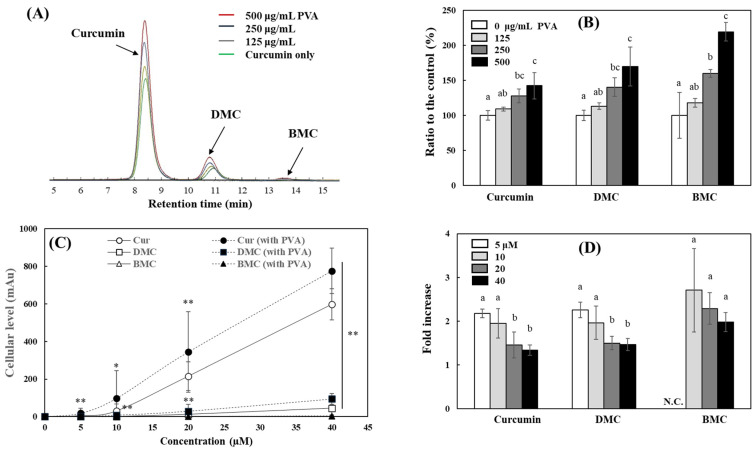
Modulation of cellular uptake of curcuminoids by PVA. INT 407 cells were incubated with 20 μM curcumin in the presence of different concentrations of PVA (0–500 μg/mL) for 90 min, and individual curcuminoid levels in cells were analyzed using the HPLC. Representative chromatograms in the current HPLC system (**A**) and relative intracellular curcuminoid levels (**B**) treated with different concentrations of PVA were shown. INT 407 cells were also incubated with different concentrations of curcumin (0–40 μM) in the presence of PVA (250 μg/mL) for 90 min, and intracellular individual curcuminoid levels (**C**) and relative levels (a fold increase based on each control) (**D**) were analyzed. Each value represents the mean ± SD (*n* = 4–6). Different letters indicate a significant difference (*p* < 0.05) based on one-way ANOVA and Tukey’s HSD test (**B**,**D**). *, **, significantly different from control according to Student’s *t*-test (*, *p* < 0.05; **, *p* < 0.01, in (**C**)).

The enhancement of curcumin activity by PVA in cell culture systems is probably due to PVA facilitating the uptake of curcumin into the cells. In the following experiment, the effects of PVA on the intracellular levels of curcumin were analyzed in curcumin-treated cells. For this purpose, INT 407 cells were treated with curcumin in the presence of different concentrations of PVA for 90 min. Since the curcumin used in the current study was a mixture of 79.4 ± 2.0, 16.8 ± 0.9, and 3.8 ± 0.7% (w/w) of curcumin, DMC, and BMC, respectively, the individual cellular levels of the three curcuminoids were analyzed by HPLC; chromatograms of these three curcuminoids in the current HPLC system are shown (Figure 6A). The intracellular uptake of curcumin, DMC, and BMC tended to increase significantly in the presence of PVA. At 250 μg/mL PVA, the cellular levels of curcumin, DMC, and BMC were significantly increased by approximately 28, 40, and 60%, respectively; the levels were further enhanced by 42, 70, and 120% in the presence of 500 μg/mL PVA (Figure 6B). The increasing effect of PVA on the intracellular levels of DMC and BMC was more pronounced than that of curcumin. Since DMC and BMC are more hydrophobic than curcumin and have more limited solubility in an aqueous system, the ability of PVA to disperse DMCs and BMCs in the cell culture media appears to have been more effective.

The effects of PVA on the cellular uptake of curcumin in the cells treated with different concentrations of curcumin were also investigated. PVA significantly increased the cellular levels of all curcuminoids in the cells treated with 5–40 μM of curcumin (Figure 6C). The enhanced cellular uptake of curcumin by PVA was more pronounced in the cells treated with lower concentrations of curcumin (Figure 6D). While PVA increased the intracellular levels of curcumin and DMC by 33–46% in cells treated with 40 µM curcumin, the effect of PVA was more than doubled in cells treated with 5 µM curcumin, with an increase in curcumin and DMC levels of >100%. The present results indicate that PVA increased the chemical stability of curcumin in an aqueous system and improved the dispersion status of curcumin in a cell culture medium. Accordingly, PVA increases the contact of curcumin with cells and enhances the uptake efficiency of curcumin by cells. This increased efficiency of curcumin uptake by PVA is thought to contribute to the enhanced bioactivity of curcumin with increased intracellular levels. With regard to the interaction of curcumin with PVA in aqueous solution, the long-chain linear form of PVA is believed to interact with curcumin through hydrophobic interactions and hydrogen bonding rather than entrapment of curcumin by nanoparticle or micelle formation, as shown in the previous studies [23,24,25,26]; PVA reduces the direct interaction of curcumin with water molecules and increases the dispersion of curcumin in solution. In doing so, PVA appears to act as a bridge, increasing the accessibility of curcumin to target molecules and cells.

## 3. Materials and Methods

### 3.1. Chemicals and Cell Lines

Curcumin, a mixture of curcumin, DMC, and BMC with content of 79.4, 16.8, and 3.8% (average molecular weight 361.1), respectively, was purchased from Acros Organics (Morris Plains, NJ, USA). The curcumin stock solution was prepared by using dimethyl sulfoxide (DMSO) at a concentration of 50 mM. PVA (partially hydrolyzed, Sigma-Aldrich, St. Louis, MO, USA) was dissolved by heating to 60 °C in DW at a concentration of 100 mg/mL, then aliquoted and frozen at −20 °C. All other chemicals and materials were obtained from Sigma-Aldrich Chemical Co. INT 407 (HeLa derived) human immortalized embryonic intestinal cells (CCL-6) and HCT 116 human colon adenocarcinoma cells (CCL-247) were obtained from the American Type Culture Collection (Manassas, VA, USA). Cell culture media and fetal bovine serum (FBS) were obtained from Welgene Inc. (Gyeonsan, Republic of Korea). INT 407 and HCT 116 cells were maintained in Eagle’s minimum essential medium (MEM) supplemented with 1% non-essential amino acids and Roswell Park Memorial Institute (RPMI) 1640 medium, respectively, containing 10% FBS, 1% antibiotics (100 unit/mL penicillin, 0.1 mg/mL streptomycin). Both cells were maintained at 37 °C, 95% humidity, and 5% CO_2_.

### 3.2. Analysis of Spectral Property and Photostability

**Figure 7:**
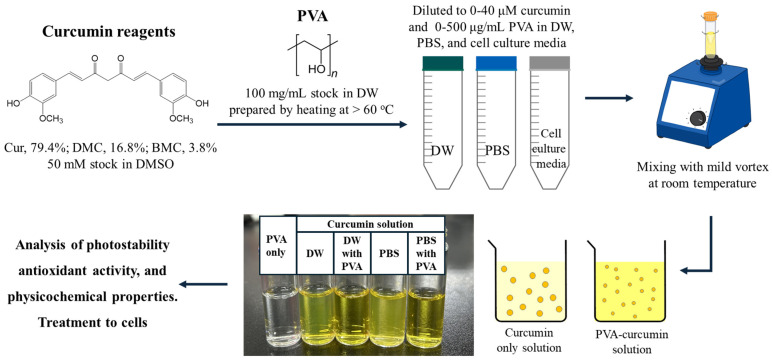
Experimental scheme for preparing curcumin–PVA solution in the present study.

A general scheme for the preparation of a solution containing curcumin and PVA is shown in Figure 7. Curcumin (40 μM) was dissolved in DW or PBS in the presence of different concentrations of PVA (0–500 μg/mL), and changes in the absorbance spectrum of curcumin by PVA were scanned at 350–700 nm using a microplate reader (SpectraMax M2). The fluorescence emission spectrum of curcumin solution was also analyzed at 450–700 nm (Ex. 440 nm) (SpectraMax M2). In addition, the change in the peak absorbance of curcumin with PVA concentration was also measured at 420 nm. To measure the change in chemical stability, the solution prepared by the above method was stored at room temperature (RT), and the absorbance change at 420 nm was measured for 24 h. For evaluation of the photostability of curcumin solutions, an ordinary household fluorescent lamp (27 W, FPL-27W EX-D) with at least three emission peaks, including 440, 550, and 620 nm, and a green (peak emission at 518 nm), white, and blue (peak emission at 447 nm) LED irradiation device (Bissol LED Co., Seoul, Republic of Korea) were used. The characteristics, including the emission spectra of the light sources, are shown in Figure S1. Curcumin (40 μM) solutions dissolved in DW containing different concentrations of PVA or PEG (0–500 μg/mL) were prepared, and 200 μL of the solution was added to each well of a 96-well plate. The plate was irradiated under each light source (10 W/m^2^ each) for 8 h at 25 °C, and photodegradation of curcumin was detected at 420 nm at each time point using a microplate reader (Spectra Max M2, Molecular device, Sunnyvale, CA, USA).

### 3.3. Measuring Antioxidant Activity

The scavenging activity of DPPH radical was determined using the method of Blois [32]. A mixture of 100 µL of 600 µM DPPH MeOH solution with 100 µL of curcumin dissolved in MeOH or DW was reacted for 30 min in the dark at RT, and absorbance was measured at 517 nm (SpectraMax M2). To analyze the ABTS radical scavenging activity, ABTS radical was prepared according to the previous method [39]. The reaction mixture of ABTS radical solution (150 μL) with curcumin dissolved in different concentrations of PVA (50 μL) was reacted for 30 min at RT and then absorbance changes were measured at 734 nm (SpectraMax M2). The measurement of NO scavenging activity was performed by the previous method [40] with a slight modification. The reaction mixture containing 100 μL of 100 μM NaNO_2_ and 50 μL of curcumin dissolved in different concentrations of PVA was incubated in a dark place at 37 °C. After 1 h, the reaction mixture was added to 50 μL of 1% sulfanilamide in 5% H_3_PO_4_ and 50 μL of 0.1% N-1-naphthyl ethylenediamine dihydrochloride (NED) solution and was incubated further for 10 min. Absorbance was measured at 540 nm. Oxygen radical antioxidant capacity (ORAC) assay was performed by the previous method [41]. Then, 60 μL of curcumin and PVA was mixed with 40 μL of 100 mM AAPH and 100 μL of 50 nM fluorescein sodium salt in PBS. A change in the fluorescence intensity of the reaction mixture was detected at Ex. 485 nm, Em. 535 nm (SpectraMax M2).

### 3.4. Measuring Physicochemical Properties of Curcumin Solution

To evaluate the dispersion stability, the DW solutions containing 40 µM curcumin with different concentrations of PVA (0–250 µg/mL) were prepared and centrifuged (1730 MR, Labogene, Daejeon, Republic of Korea) at 14,000 RCF for 15 min. After the centrifugation, 200 µL of the supernatant was collected, and the absorbance was measured at 420 nm. The remaining pellet was dissolved with 200 µL of DMSO, and 180 µL of this solution was used to measure the absorbance at 420 nm. To investigate the effect of PVA on the viscosity of DW and RPMI 1640, different concentrations of PVA were added to each solution without curcumin or in the presence of 20 µM curcumin. The solutions were injected in amounts of 5 mL each into a calibrated viscometer (CFRC-150, Cannon Instrument Company, State College, PA, USA), and the relative viscosities were compared by measuring the flow time at 25 °C. The particle size in curcumin solutions was also measured in order to assess the degree of dispersion status. Different concentrations of PVA and 20 µM curcumin were dissolved in DW, PBS, and serum-free RPMI 1640 media with antibiotics. Each sample solution (750 µL) was injected into a cuvette and the particle (droplet) size was measured using a Zeta potential analyzer (DTS1070, Malvern Instruments Ltd., Worcestershire, UK).

### 3.5. Analyzing Cytotoxic Activity

The cytotoxic effects of curcumin were determined using a 3-(-4,5-dimethylthiazol-2-yl)-2,5-diphenyltetrazolium bromide (MTT) assay. INT 407 and HCT 116 cells were seeded at 1.5 ×10^4^ cells/well in 96-well plates in a growth media. Each cell was treated the next day with curcumin dissolved in serum-free media in the absence or presence of PVA. After 24 h treatment, the treated medium was removed, and a fresh medium containing 0.5 mg/mL MTT was added to each well. The cells were further incubated at 37 °C for 1 h. The medium was then removed and the MTT formazan formed in cells was solubilized with 100 µL of DMSO; the absorbance was measured at 550 nm using a microplate reader (Spectra Max M2).

### 3.6. Measuring Intracellular Uptake of Curcumin

INT 407 cells were seeded in a 6-well plate at 1 × 10^6^ cells/well using a growth medium. When ~80% confluency was reached, the cells were treated with curcumin dissolved in serum-free media in the absence or presence of PVA (0–500 mg/mL). After 1.5 h of incubation, the cells were washed 3 times with ice-cold PBS and lysed with 70% MeOH. The cell lysates were centrifuged at 10,000 RCF for 10 min at 4 °C, and the supernatant was mixed with HPLC solvent (2:1, *v*/*v*). The HPLC system for analyzing curcumin consisted of an Agilent-HPLC system 1100 (Agilent Technologies, Waldbronn, Germany), Shiseido Capcellpak C18 UG120 reversed-phase column (150 × 4.6 mm; particle size, 5 µm). HPLC analysis was performed according to the previous method [42]. The mobile phase consisted of deionized water containing 1% citric acid and THF (60:40, *v*/*v*) adjusted to pH 3.0 with concentrated KOH solution and was run isocratically. The detection wavelength of curcumin was set at 420 nm. The flow rate was 1 mL/min and the injection volume was 20 µL. The detailed analytical conditions are summarized in Table S1.

### 3.7. Data Analysis

All values represent the mean ± standard deviation (SD). Statistical significance was evaluated using Student’s *t*-test. One-way analysis of variance (ANOVA) and Tukey’s honestly significant difference (HSD) test were used to compare multiple results (SPSS, version 21.0, Chicago, IL, USA).

## 4. Conclusions

The hydrophobic nature of curcumin is a major limitation that prevents many aqueous assay systems from properly measuring its activity, despite the extensive and ongoing research on curcumin compounds in light of their various health benefits. Therefore, in this study, a suitable vehicle for curcumin in an aqueous experimental system was explored to overcome these limitations. The results of the present study indicate that PVA increased the chemical and photostability of curcumin in aqueous solution and enhanced its activity in various antioxidant assay systems. PVA also improved the dispersion status and solution stability of curcumin in aqueous systems and reduced the aggregation of curcumin molecules, which might increase the opportunity for curcumin to interact with target molecules. In particular, PVA induced efficient intracellular delivery of curcumin and enhanced bioactivities of curcumin with little effect on the cells in cell culture systems. These results suggest that PVA may be a suitable vehicle for analyzing the more accurate activity of curcumin in various aqueous experimental systems. Furthermore, we believe that the results of this study have a wide range of applications and implications, as PVA can be used as a delivery material to increase the bioavailability of curcumin and its pharmaceutical efficacy in the body.

## Data Availability

Data are available from the corresponding author upon reasonable request.

## Supplementary Materials

The following supporting information can be downloaded at https://www.mdpi.com/article/10.3390/ijms25116278/s1.
