# Supplementary material for: Enhancement of Solubility, Stability, Cellular Uptake, and Bioactivity of Curcumin by Polyvinyl Alcohol
Source: Int J Mol Sci. 2024 Jun 6;25(11):6278. doi: 10.3390/ijms25116278 (PMC11172464; doi:10.3390/ijms25116278)
Supplement: Supplementary file 1 [file ijms-25-06278-s001.zip › ijms-3026706-supplementary.pdf]

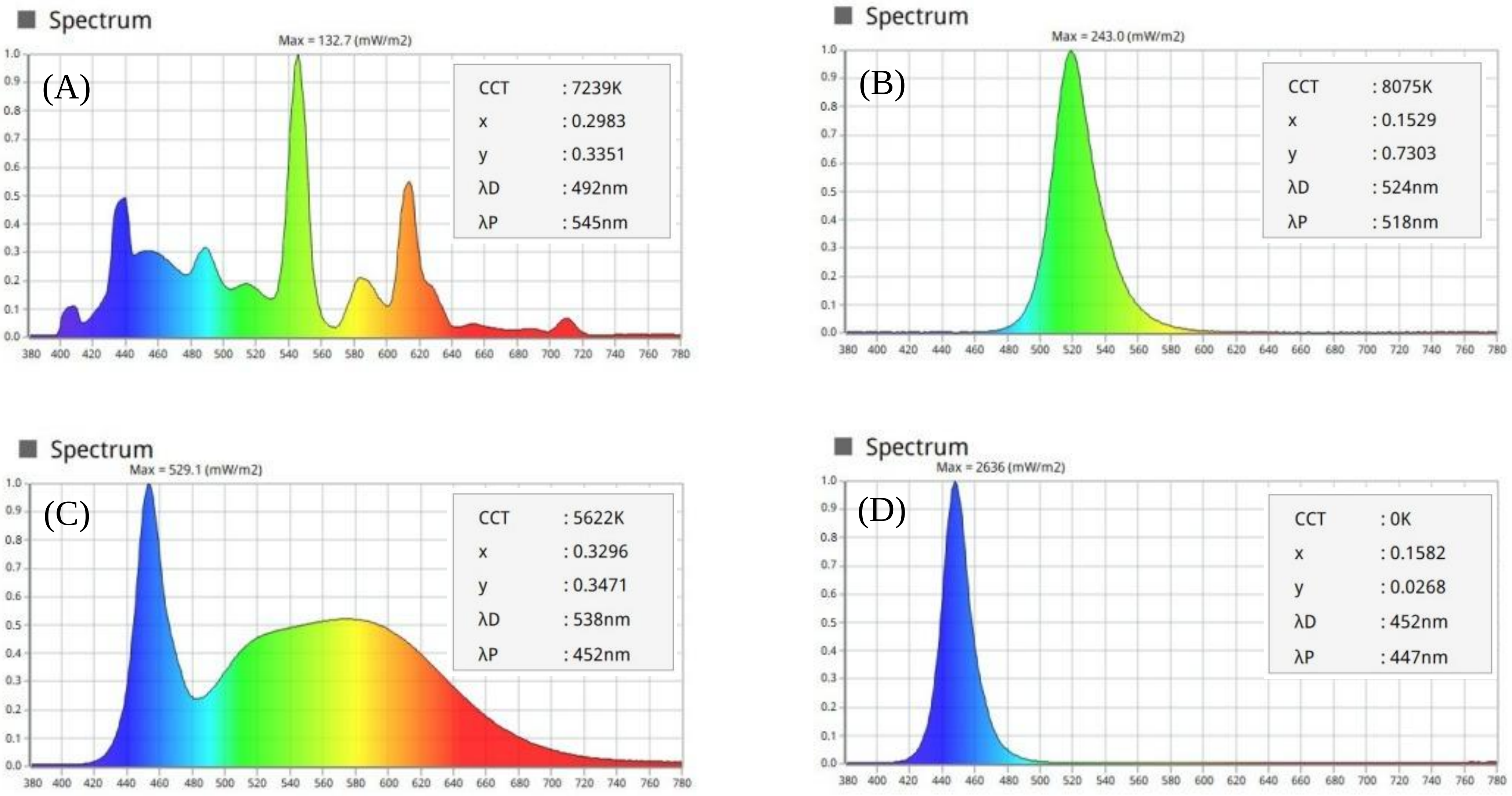

Supplementary Figure. S1 Emission spectra and characteristics of the irradiated lights including regular fluorescent light (A), green (B), white (C) and blue (D) LEDs used in the present study. CCT, color correction temperature; x and y, CIE1931 color coordinate; λD, dominate wavelength; λP, peak emission wavelength.

Supplementary Table S1. Operating conditions for analyzing individual curcuminoid in the current HPLC system

**Instrument**	Agilent 1100 series (Agilent Tech., HPS 8, Waldbronn, Germany)
**Column**	Capcellpak C_{18} UG120 (4.6 mm IDx150 mmx5 µm; Shiseido, Tokyo, Japan)
**Detector**	G1314A variable wavelength detector (VWD) with standard flow cell<br>(10 mm path length, 14 µL volume, 40 bar maximum pressure)
**Flow rate**	1 mL/min
**Injection volume**	20 µL
**Mobile phase**	40% THF:60% water:1% citric acid (v/v/v, pH 3 adjusted by concentrated KOH)
